# Lactobacillus casei DG and its postbiotic reduce the inflammatory mucosal response: an ex-vivo organ culture model of post-infectious irritable bowel syndrome

**DOI:** 10.1186/s12876-017-0605-x

**Published:** 2017-04-14

**Authors:** Debora Compare, Alba Rocco, Pietro Coccoli, Debora Angrisani, Costantino Sgamato, Barbara Iovine, Umberto Salvatore, Gerardo Nardone

**Affiliations:** Department of Clinical Medicine and Surgery, Gastroenterology Unit, University Federico II of Naples, Via S. Pansini 5, 80131 Naples, Italy

**Keywords:** Post-infectious irritable bowel syndrome, Probiotics, Postbiotic

## Abstract

**Background:**

The evidence on the role of gut microbiota in post-infectious irritable bowel syndrome (PI-IBS) is convincing. Lactobacillus spp. positively affect IBS symptoms, although the mechanisms through which probiotics exert their beneficial effects are largely unknown. The aim of the study is to evaluate the role of Lactobacillus casei DG (LC-DG) and its postbiotic (PB) in modulating the inflammatory/immune-response in PI-IBS in an ex-vivo organ culture model.

**Methods:**

Ex vivo cultures of ileal and colonic mucosa from 10 PI-IBS, diarrhea predominant subtype (D) patients, and 10 healthy controls (HC) were treated with LPS, LC-DG and PB. Interleukin (IL)-1α, IL-6, IL-8 and IL-10 mRNA levels were assessed by real-time PCR and Toll like receptor 4 (TLR-4) protein expression by Western blotting.

**Results:**

At baseline, IL-1α, IL-6 and IL-8 mRNA levels as well as TLR-4 protein expression were significantly higher while IL-10 mRNA levels were lower in PI-IBS D than in HC in both ileum and colon. LC-DG and PB significantly reduced the mRNA levels of pro-inflammatory cytokines and TLR-4 while increased that of IL-10 after LPS stimulation. The protective effect was more pronounced for PB than LC-DG treatment.

**Conclusion:**

LC-DG and its PB attenuate the inflammatory mucosal response in an ex-vivo organ culture model of PI-IBS D.

## Background

Irritable bowel syndrome (IBS) is the commonest functional gastrointestinal disorder, affecting up to 20% of the population in Europe and the USA [1]. IBS does not predispose patients to severe illness but it deeply affects the quality of life and incurs a significant economic burden in both direct and indirect expenditures worldwide [2]. According to the symptom’s pattern IBS is subcategorized into IBS with constipation (IBS-C), IBS with diarrhea (IBS-D), mixed IBS (IBS-M) and unsubtyped IBS (IBS-U) [3]. Gastrointestinal dysmotility, visceral hypersensitivity, brain-gut axis dysfunction, and alterations in psychosocial or psychosomatic behavior have been implicated in the pathophysiology of IBS, but the exact mechanisms remain largely undefined [4]. Recently, IBS research focused on causative factors such as low-grade mucosal inflammation and local immune activation [5], both triggered by perturbations of gut microbiota. The evidence for gut microbes playing a role in the pathogenesis of IBS is convincing. Small intestinal bacterial overgrowth (SIBO) is frequently detected in IBS patients and symptoms pattern of SIBO largely overlap those of IBS [6]. Recently, qualitative alterations of gut microbiota in subjects with IBS were also revealed by metagenomic approaches [7]. Based on the assumption that IBS develops in up to 30% of individuals recovering from acute gastroenteritis, the pathogenetic role of gut microbiota was well established mainly for post-infectious (PI)-IBS, diarrhea (D) predominant subtype [8]. As a consequence, the manipulation of the microbiota is becoming an attractive therapeutic option for this disease [9]. A recent systematic review focusing on the efficacy of Lactobacillus spp in IBS found a positive effect on symptom relief [10]. However, few studies have addressed the mechanisms through which probiotics exert their beneficial effects. Moreover, growing data, mainly obtained by the analysis of Lactobacilli strains, support the evidence that these beneficial effects may depend on secreted probiotic-derived factors, recently identified as postbiotic (PB) mediators [11].

## Methods

Aim of this study was to evaluate the role of Lactobacillus casei DG (LC-DG) and its PB in modulating the inflammatory immune-response in an ex-vivo organ culture model of PI-IBS D. Patients with a diagnosis of PI-IBS who met Rome III criteria for IBS-D were consecutively recruited among 92 patients with IBS referred to the Gastroenterology Unit of the University Federico II of Naples from December 2014 to June 2015. A total of 10 out of 13 PI-IBS patients (6 males; mean age 52 years) accepted to participate to the study. The patients confirmed IBS onset after an episode of acute gastroenteritis with diarrhea and/or vomiting occurred at least one year before the enrollment, thus fulfilling the definition of PI-IBS [12]. Ten control subjects (5 males; mean age 48 years) were recruited among those referred to perform a colonoscopy indicated within the framework of colorectal cancer screening (age, anemia, positivity of the fecal occult blood test, rectal bleeding, personal or family history of polyps, family history of colorectal cancer). Inclusion criteria were age ≥18 and ≤70 years and normal findings at endoscopic and histological examinations. Presence of any of the following criteria excluded patients from the study: ascertained inflammatory bowel diseases (Crohn’s disease, diverticular disease, ulcerative colitis, ischemic colitis, microscopic colitis, coeliac disease), topic or systemic antibiotic and probiotic therapy during the last month, therapy with nonsteroidal anti-inflammatory drugs, proton pump inhibitors or H2-antagonists, antiplatelet and/or anticoagulant drugs, intended or ascertained pregnancy or lactation, copro-parasitological examination of stools positive, active malignancy of any kind, or a history of a malignancy, clinically relevant renal, hepatic, haematologic, cardiac, neurological, psychiatric, immunological, gastrointestinal, metabolic or endocrine disease, abuse of alcohol, drugs or psychotropic drugs which may affect alertness and physical perception and inability to conform to the protocol or denied consensus. All subjects underwent ileo-colonoscopy with multiple bioptic sampling at ileal and left colon sites for both histology and ex vivo organ culture.

### Probiotic and postbiotic preparation

Lactobacillus Casei DG was obtained dried by SOFAR (Milan, Italy) and stored at a temperature below 25 ° C until use. Bacteria were restarted at 1:100 and grown in MRS broth (Biokar Diagnostic, Beauvais Cedex, France) to an OD600 = 0.6. Bacterial cultures were plated to count effective colony forming units (CFUs). The growth curve was evaluated after 24 h at 37 °C in conventional bacterial incubation or in a 5% CO2 incubator for eukaryotic cells or in oxygen chamber, which was filled with pressurized oxygen. The growth profile was determined by the calculation of the specific growth rate (u) that is the change in the number of cells in unit of time u = (OD1/OD2)/(T2-T1). Postbiotic was obtained by centrifugation at 10,000xg for 15′ of a LC-DG culture in exponential phase with equivalent amount of CFUs.

### Experimental procedures

**Fig. 1:**
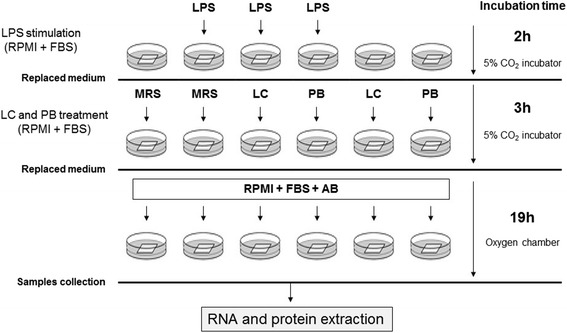
Schematic diagram of the experimental procedures

The experimental procedures are summarized in Fig. 1. Mucosal bioptic samples from ileum and left colon were immediately placed on culture filter plates (15 mm diameter wells with 500 mm bottom-mesh, Netwell culture system, Costar, Cambridge, MA, USA) with the epithelial surface uppermost. Filters were placed into wells containing 1 ml RPMI (Gibco Laboratories, North Andover, MA), 10% fetal bovine serum (FBS) (Gibco Laboratories, North Andover, MA, USA) in presence or absence of 100 μg/ml lipopolysaccharides (LPS) (Sigma-Aldrich, Milan, Italy), and without antibiotics for 2 h at 37 °C in a 5% carbon dioxide incubator. Then, the medium was substituted with RPMI with 10% FBS, containing LC-DG (1x107 CFU) or the PB or equal volume of unfermented bacterial medium MRS medium as control. After 2 h at 37 °C in a 5% CO2 incubator, the medium was removed and replaced with RPMI 10% FBS, containing 3% penicillin/streptomycin (Gibco Laboratories, North Andover, MA, USA) and 50 μg/ml gentamycin. The tissues were then transferred into the oxygen chamber, which was filled with pressurized oxygen (VitalAire, Milan, Italy) and placed at 37 °C for the remaining 19 h of culture. The control samples were treated in the same way without addition of LPS, LC-DG or PB.

### RNA and protein extracts preparation

Total RNA and protein extracts were prepared from intestinal biopsies by using the TRIzol Reagent (Life Technology, Carlsbad, CA, USA). Briefly, each biopsy was placed in 200 μl of TRIzol Reagent and homogenized using a glass Teflon. Following homogenization the samples were centrifuged at 12,000 g for 10′ at 4 °C and the aqueous phase, containing RNA, and the organic interphase, containing proteins, were processed separately according to the data sheet protocol. Total RNA quantity and quality were evaluated by RNA nanodrop and displaying on denaturing agarose gel. RNAs were stored at −80 °C until use for Real Time.

### Real-time PCR

1 μg total RNA was used to synthesize the cDNA according to the iScript cDNA Synthesis kit protocol (Bio-Rad Laboratories, Hercules, CA, USA). The cDNA was then amplified in an iCycler iQ real time PCR detection system (Bio-Rad Laboratories, Hercules, CA, USA) by using iQTM SYBR Green Supermix (Bio-Rad Laboratories, Hercules, CA, USA). The Real-Time PCR reactions and the relative quantification gene expression were performed as previously reported [13]. Each experiment was performed in triplicate. Relative quantification of gene expression was performed using the 2-∆∆CT method. The primer sequences of the analyzed interleukins (ILs) are reported in Table 1.

**Table 1:** Primers used for real time PCR experiments

IL-1α	F:5′-CGCCAATGACTCAGAGGAAGA-3′ R:5′-AGGGCGTCATTCAGGATGAA-3′
IL-6	F:5′-TACCCCCAGGAGAAGATTCC-3′ R:′-GCCATCTTTGGAAGGTTCAG-3′
IL-8	F:5′-AGACAGCAGAGCACACAAGC R:5′-ATGGTTCCCCTTCCGGTGGT-3′
IL-10	F:5′-GAACCAAGACCCAGACATC-3′ R:5′-CATTCTTCTTCACCTGCTCCAC-3′

### Western blotting analysis

Protein concentrations were determined by a Bio-Rad protein assay (Bio-Rad Laboratories, Hercules, CA, USA) and 20 μg were diluted with loading buffer and heated to 95 °C for 10′. Then, protein lysates were run onto 10% SDS-PAGE gel and transferred on PVDF membrane (MILLIPORE). Membranes were probed overnight at 4 °C with rabbit anti-TLR4 (1:1,000; Novus, Littleton, CO, USA) and rabbit anti-α-actin (1:1,000; Santa Cruz Biotechnology, Dallas, TX) antibodies. Each experiment was performed in triplicate. The signals were detected after the incubation with anti-rabbit peroxidase-conjugated secondary antibodies (1:5,000; Santa Cruz Biotechnology, Dallas, TX, USA) by using the ECL kit (Advansta, Menlo Park, CA, USA). The band densities were assessed using Image J 1.40 g software.

### Statistical analysis

All data collected were summarized separately for each patient in all experimental conditions and expressed as mean ± standard deviation. Mann–Whitney *U* test and one-way ANOVA, when appropriate, were used to compare the variables. A *p* value < 0.05 was set as level of significance. Statistical analysis was performed with SPSS for Windows (version 19.0; IBM Corporation, Armonk, NY, USA). Prism for Windows 5 (version 5.02; GraphPad Software Inc., La Jolla, CA, USA) was used for graphical presentation.

## Results

### Effect of LC-DG and PB on IL-1α, IL-6, IL-8 mRNA and IL-10 mRNA levels

**Fig. 2:**
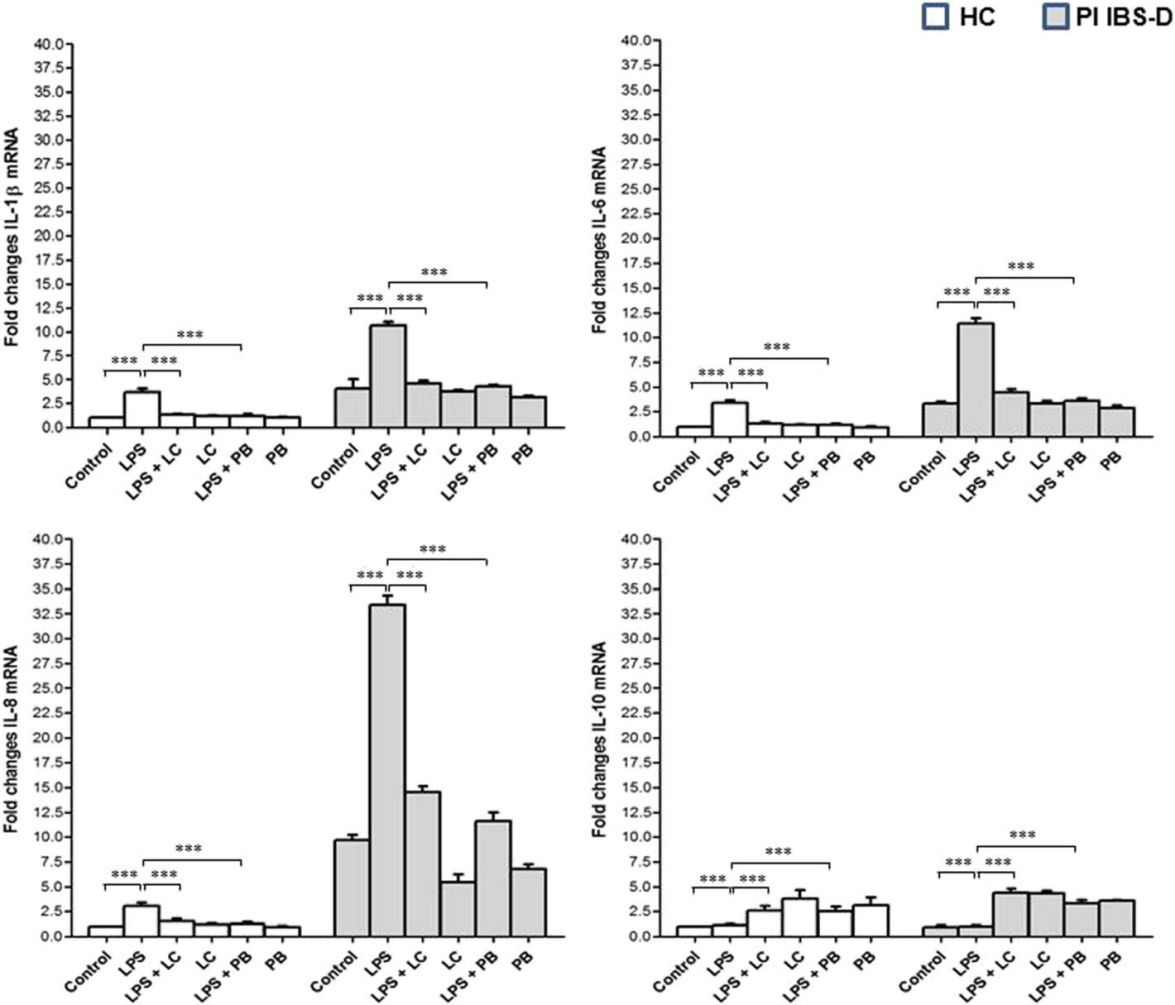
Fold changes in mRNA levels of IL-1α, IL-6, IL-8 and IL-10 in the ileal mucosa of HC and IBS-D patients. IL-1α, IL-6 and IL-8 mRNA baseline levels were higher and IL-10 mRNA levels were lower in post-infectious IBS-D than HC. The stimulation of intestinal mucosa with 100 μg/ml LPS significantly increased mRNA levels of all cytokines in respect to baseline in both HC and PI-IBS D patients. In contrast, LPS treatment did not affect IL-10 mRNA levels in both HC and IBS-D. LC-DG treatment was effective in reducing IL-1α and IL-8 mRNA levels and increasing IL-10 m-RNA levels. PB treatment was effective in reducing IL-1α, IL-6 and IL-8 mRNA levels and increasing IL-10 m-RNA levels. ***p < 0.0001. HC: healthy controls; PI IBS-D: post-infectious irritable bowel disease diarrhea subtype; LPS: lipopolysaccharide; LC: Lactobacillus Casei DG; PB: postbiotic

**Fig. 3:**
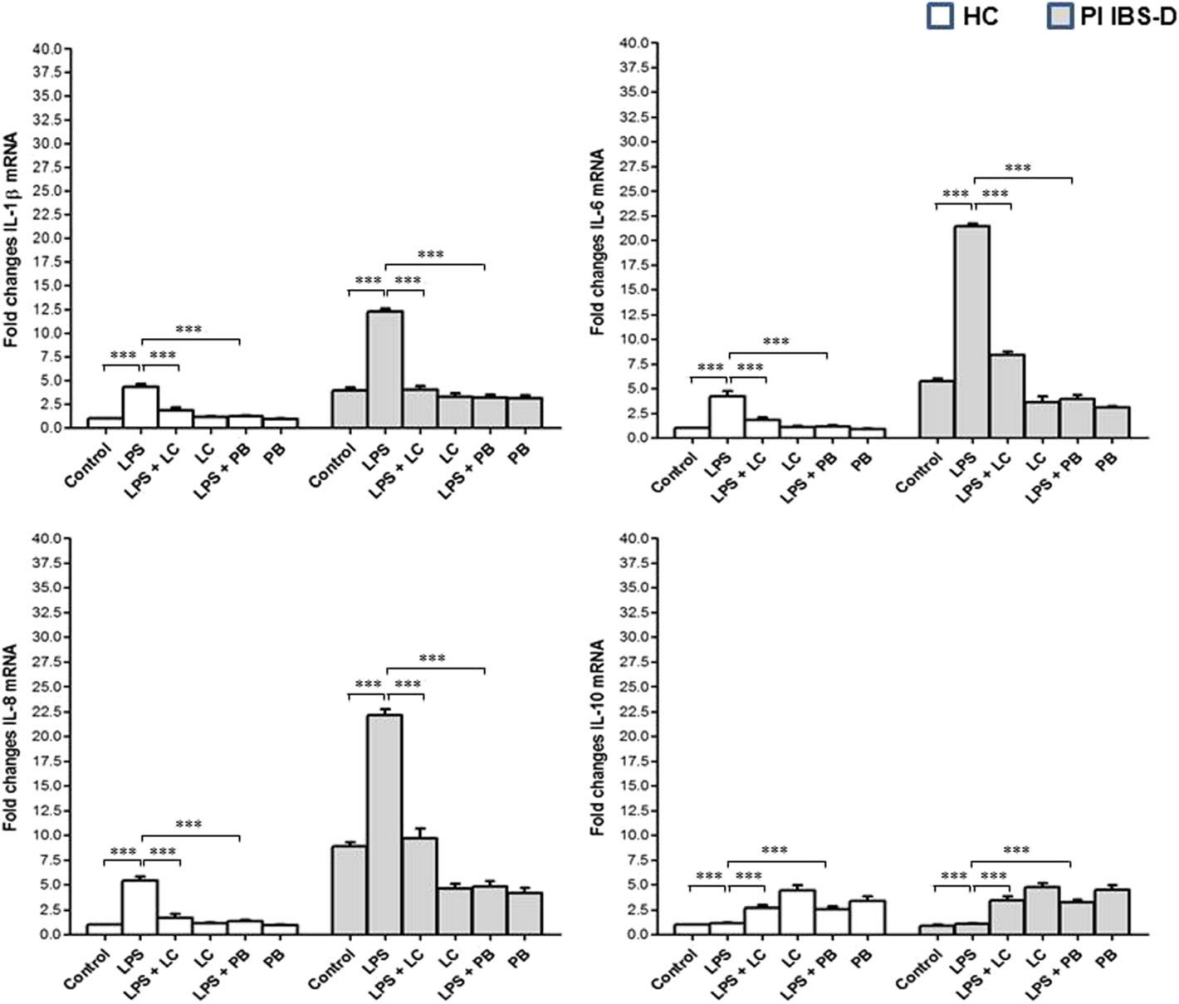
Fold changes in mRNA levels of IL-1α, IL-6, IL-8 and IL-10 in the left colon mucosa of HC and IBS-D patients. IL-1α, IL-6 and IL-8 mRNA baseline levels were higher and IL-10 mRNA levels were lower in PI-IBS D than HC. The stimulation of intestinal mucosa with 100 μg/ml LPS significantly increased mRNA levels of all cytokines in respect to baseline in both HC and PI-IBS D patients. In contrast, LPS treatment did not affect IL-10 mRNA levels in both HC and IBS-D. LC-DG and PB treatment were effective in reducing IL-1α, IL-6 and IL-8 mRNA levels and increasing IL-10 m-RNA levels. ***p < 0.0001. HC: healthy controls; PI IBS-D: post-infectious irritable bowel disease diarrhea subtype; LPS: lipopolysaccharide; LC: Lactobacillus Casei DG; PB: postbiotic

At baseline, IL-1α, IL-6 and IL-8 mRNA levels were higher while IL-10 mRNA levels were lower in PI-IBS D than HC, irrespective of intestinal mucosa site (Figs. 2 and 3). Notably, in PI-IBS D patients, IL-6 mRNA levels were higher in colonic than in ileal mucosa while IL-8 mRNA levels were higher in ileal than in colonic mucosa. The stimulation of intestinal mucosa with 100 μg/ml LPS significantly increased mRNA levels of all cytokines in respect to baseline in both HC and PI-IBS D patients (Figs. 2 and 3). However, the magnitude of the inflammatory response of the intestinal mucosa, that is the difference between LPS-induced mRNA levels and baseline values, was greater in patients than in HC in both ileal and colonic mucosa (Il-1α *p* < 0.0001, IL-6 *p* < 0.0001 and IL-8 *p* < 0.0001). In contrast, the magnitude of the anti-inflammatory response did not significantly differ between HC and IBS-D, irrespective of mucosal site.

In PI-IBS D, the treatment of colonic biopsies with LC-DG significantly reduced the levels of all pro-inflammatory cytokines (Il-1 α *p* < 0.002, IL-6 *p* < 0.0001 and IL-8 *p* < 0.0001) in respect to baseline. In ileal mucosa, LC-DG treatment was effective in reducing IL-1α and IL-8 mRNA levels (*p* < 0.0002 and *p* < 0.0001, respectively) but did not affect IL-6 mRNA levels. LC-DG treatment significantly increased IL-10 m-RNA levels in both colonic and ileal mucosa (*p* < 0.0001 and *p* < 0.0001, respectively). Similarly, PB treatment was effective in reducing IL-1α, IL-6 and IL-8 mRNA levels in both colonic (*p* < 0.0001, *p* < 0.0001 and *p* < 0.0001, respectively) and ileal mucosa (*p* < 0.0001, *p* < 0.0006 and *p* < 0.0001, respectively). In contrast, IL-10 m-RNA levels significantly increased in both ileal and colonic mucosa (*p* < 0.0001 and *p* < 0.0001, respectively). The protective effect of LC-DG and PB was not affected by the pre-treatment of intestinal biopsies with LPS. Interestingly, the effect was more pronounced for PB treatment in respect to LC-DG treatment, in all cases.

### Effect of LC-DG and PB on TLR-4 protein expression after LPS stimulation

**Fig. 4:**
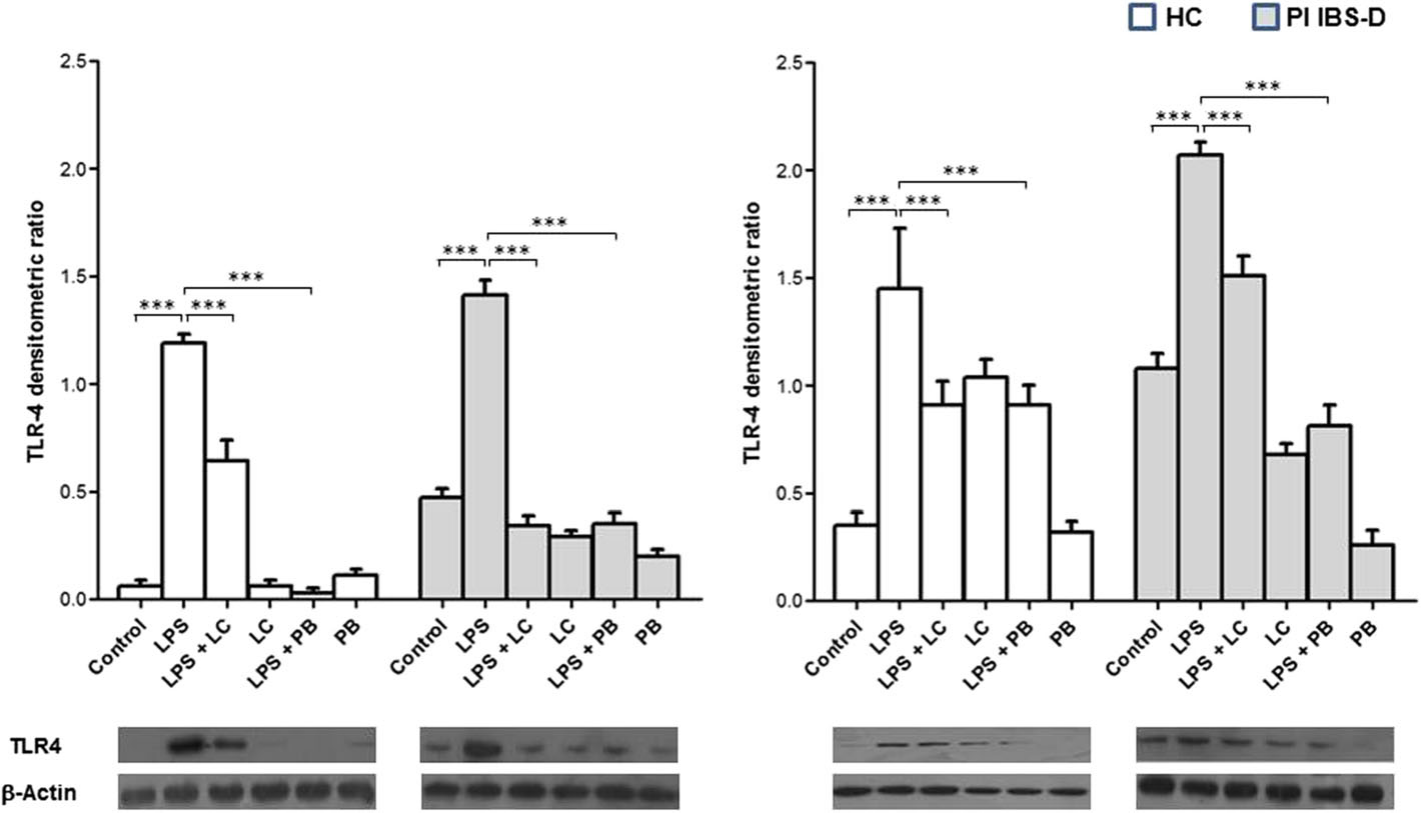
TLR-4 protein expression in ileal and left colon mucosa of HC and IBS-D patients. TLR-4 protein baseline levels were higher in PI-IBS D patients in respect to HC in both ileal and colonic mucosa. The stimulation of intestinal mucosa with 100 μg/ml LPS increased TLR-4 protein expression in both HC and IBS-D patients with a more pronounced effect in colonic mucosa. LC-DG and PB treatment reduced TLR-4 protein expression in both HC and IBS-D patients particularly in ileal mucosa. ***p < 0.0001. HC: healthy controls; PI IBS-D: post-infectious irritable bowel disease diarrhea subtype; LPS: lipopolysaccharide; LC: Lactobacillus Casei DG; PB: postbiotic

At baseline, TLR-4 protein expression was significantly higher in PI-IBS D patients in respect to HC in both ileal and colonic mucosa. In details, TLR-4 protein expression was 7.4-folds higher in ileal mucosa (*p* < 0.0001) and 3-folds higher in colonic mucosa (*p* < 0.001) of PI-IBS D patients as compared with HC. LPS stimulation significantly increased TLR-4 protein expression in both HC and IBS-D patients (*p* < 0.0001) with a more pronounced effect in colonic mucosa. The increase of TLR-4 protein expression was attenuated by LC-DG and PB treatment in both HC and IBS-D patients (*p* < 0.0001). Interestingly, the protective effect was more evident in ileal than in colonic mucosa (Fig. 4).

## Discussion

In our study, the baseline expression of pro-inflammatory cytokines, namely IL-1α, Il-6 and Il-8 was significantly higher and that of IL-10 significantly lower in the intestinal mucosa of PI-IBS D patients in respect to HC.

An increased number of lamina propria lymphocytes and mRNA levels of pro-inflammatory cytokines with a parallel decrease of mRNA levels of anti-inflammatory cytokines have been described in colonic mucosa of PI-IBS as compared to non-PI-IBS and control subjects [14, 15].

Moreover, PI-IBS patients had an increased expression of IL-1α mRNA in terminal ileum and recto-sigmoidal mucosa [16]. These data support the hypothesis that the immune dysfunction and the dysregulated neuroimmune interactions may predispose individuals to IBS [8]. Based on this pathophysiological model an event, such as infection, would generate permanent disturbances of the gut microbiota with overgrowth of pathogens and a marked reduction in bacterial diversity. The breakdown of the microbial ecology might be responsible for the “chronic low grade inflammation” of the intestinal mucosa, typically observed in IBS patients. In addition, an altered host-microbiota interaction, may also contribute to the pathophysiology of IBS.

TLRs represent a first line of host defense to pathogens by activating responses in cells of the innate immune system [17]. Brint et al. firstly reported a 4-fold increase in the TLR-4 mRNA expression in recto-sigmoid mucosa of IBS patients as compared with controls [18]. However, this study did not analyze the right colon and terminal ileum mucosa, the site of highest concentrations of bacteria and where most immunological events occur. Moreover, even if PI-IBS could represent the most relevant subtype for the assessment of TLR expression, due to the infectious origin, this subgroup of patients was excluded from the study. In our study, TLR-4 protein expression was significantly higher in PI-IBS D patients in respect to HC, in both ileal and colonic mucosa. Interestingly, while in the HC group the TLR-4 protein expression did not significantly differ between ileal and colonic mucosa, in PI-IBS D patients we found a significantly increased TLR-4 protein expression in the ileal mucosa.

Our data are in line with both basic and clinical evidence on the causative role of intestinal dysbiosis in the pathogenesis of IBS, thus supporting the idea that the modulation of gut microbiota could be an attractive treatment option for the disease. Randomised controlled trials demonstrated that probiotics were effective in the modulation of symptom’s pattern of IBS patients [19–24]. However, the majority of the studies have been performed in non-specific IBS rather than in PI-IBS D, so that the exact role of probiotics in the management of these patients remains to be elucidated. In addition, the subjective nature of the symptoms used as surrogate end-points in clinical trials does not prove the “anti-inflammatory” effect of probiotics in IBS. On the other hand, the assessment of the effect of probiotics on intestinal mucosa would require repeated endoscopic examinations with extensive bioptic sampling, that is unfeasible because of patient acceptability and ethical concerns. Probiotics action has been studied on isolated cells and cell lines, cell co-cultures and mouse models [25–27]. These models do not accurately represent the unique microenvironment of the intestine as they all lack important human-specific components like the mucus and the microbiota [27]. The development of a model system that resembles the human intestine is therefore of great value for testing the action of probiotics on both healthy and diseased tissues. The use of entire highly viable human intestinal biopsy specimens in culture is the ideal model, especially to analyze biological phenomena occurring within the first 24–48 h after which the epithelium in the biopsy specimens is no more viable [11]. By using such a model we analysed the effect of the probiotic strain LC-DG on ileal and colonic mucosa of PI-IBS D patients. The strain we used in the experimental setting was selected on the basis of its human origin, non-pathogenicity, resistance to intestinal acid and bile, ability to adhere to human epithelial cells and colonize the human gastrointestinal tract. Several studies found a decrease of Lactobacilli spp. in faeces and mucosa of IBS patients [28–30], while, interventional trials demonstrated the capability of Lactobacilli to improve symptoms and modulate the inflammatory response in these patients [10]. However, to the best of our knowledge, no data are available on the effect of LC-DG on the mucosal inflammatory response. In our study, we demonstrated that treatment of intestinal biopsies with LC-DG significantly reduced mRNA levels of all pro-inflammatory cytokines as well as the TLR-4 protein expression while increased the mRNA levels of the anti-inflammatory IL-10 in both ileal and colonic mucosa of PI-IBS D patients. Interestingly, the pretreatment of intestinal biopsies with LPS did not affect the anti-inflammatory action of LC-DG.

Probiotics act through molecular and cellular mechanisms that contrast pathogen bacteria adhesion,

enhance innate immunity, decrease pathogen-induced inflammation, and promote intestinal epithelial cell survival, barrier function, and protective responses. However, Lactobacilli-derived PB mediators seem to mediate the beneficial effects of probiotics [11]. Interestingly, in our study, the modulatory effect on inflammatory response of the intestinal mucosa was even more successful when we used the PB obtained from LC-DG cultures.

We are aware that the relatively small number of patients included in our study prevents us to generalize our results. However, strengths of our study are the rigorous selection of the patients, the “ex vivo” organ culture model we used, the sampling of both ileal and left colon mucosa and the analysis of the effect of both the probiotic strain LC-DG and its PB on the inflammatory response of the intestinal mucosa.

## Conclusions

LC-DG and its PB attenuate the inflammatory mucosal response in an ex-vivo organ culture model of PI-IBS D. These findings provide biological plausibility to the therapeutically usefulness of this probiotic strain in the clinical setting of PI-IBS D. Well designed and powered clinical trials are advisable to confirm the therapeutic efficacy in the real life management of these patients.

## Abbreviations

FBS: Fetal bovine serum
HC: Healthy controls
IBS: Irritable bowel syndrome
IBS-C: Irritable bowel syndrome IBS with constipation
IBS-D: Irritable bowel syndrome IBS with diarrhea
IBS-M: Mixed irritable bowel syndrome
IBS-U: Unsubtyped irritable bowel syndrome
IL: Interleukin
LC-DG: Lactobacillus casei DG
LPS: Lipopolysaccharides
LPS: Postbiotic
PI-IBS D: Post infectious irritable bowel syndrome diarrhea predominant subtype
PI-IBS: Post-infectious irritable bowel syndrome Irritable bowel syndrome
SIBO: Small intestinal bacterial overgrowth
TLRs: Toll like receptors
